# One-Stage High-Density Focal Stereo-Array SEEG-Guided Radiofrequency Thermocoagulation for the Treatment of Pediatric Giant Hypothalamic Hamartomas

**DOI:** 10.3389/fneur.2020.00965

**Published:** 2020-09-02

**Authors:** Min Wang, Yuanfeng Zhou, Yi Zhang, Wei Shi, Shuizhen Zhou, Yi Wang, Hao Li, Rui Zhao

**Affiliations:** ^1^Department of Neurosurgery, Children's Hospital of Fudan University, National Children's Medical Center, Shanghai, China; ^2^Department of Neurology, Children's Hospital of Fudan University, National Children's Medical Center, Shanghai, China

**Keywords:** children, stereoelectroencephalography, hypothalamic hamartoma, radiofrequency thermocoagulation, treatment

## Abstract

**Background:** Giant hypothalamic hamartomas (HHs) are extremely rare lesions, for which the treatment is challenging. While minimally invasive treatments such as radiofrequency thermal coagulation and laser ablation have improved seizure outcomes, multiple operations are often required. This study investigated the value of one-stage stereo-array radiofrequency thermocoagulation based on stereotactic electroencephalography (SEEG) for pediatric giant HHs.

**Methods:** We analyzed the clinical data of six patients with giant HHs (masses with a maximum diameter >30 mm) who underwent stereotactic electrode implantation between November 2017 and April 2019. After a multidisciplinary discussion, we designed a high-density focal stereo-array electrode implantation strategy. SEEG-guided bipolar coagulations were performed between two contiguous contacts of the same electrode, or between two adjacent contacts of different electrodes.

**Results:** Among the six patients, three were male and three were female, with an average age of 5.08 ± 4.73 years (range, 1.4–12 years); the average follow-up duration was 20.17 ± 5.49 months. One patient had previously undergone open surgery. Four patients had gelastic seizures, one had gelastic and tonic seizures, and one had gelastic and generalized tonic-clonic seizures. The number of implanted electrodes ranged from 3 to 7, with an average of 5.33. One patient had transient diabetes insipidus after the operation, and no child had fever or new hormone metabolisms disorder after surgery. Four patients had Engel I classification outcomes (free from disabling seizures), and two patients had Engel II classification outcomes.

**Conclusion:** Although the exploration of epileptic activity and the extent of ablation are limited by the number of SEEG electrodes for the complete disconnection. One-stage high-density focal stereo-array SEEG-guided radiofrequency was safe and effective for treating pediatric giant HH patients. It can be an alternative method to treat giant HHs where LITT is unavailable.

## Introduction

Hypothalamic hamartomas (HHs) are rare congenital abnormalities (1) that typically manifest as refractory epilepsy (2) and are located in the region of the tuber cinereum and the third ventricle (1). Other manifestations may include central precocious puberty and developmental retardation (3–5). HHs are non-neoplastic heterotopias that consist of normal neuronal tissue (1). The estimated incidence of HH is one per 100,000–1,000,000 (6). Giant HHs are even more rare, and to the best of our knowledge, only sporadic cases have been reported in childhood (5, 7–12). In contrast to the previously accepted hypothesis that epileptogenesis is strictly a cortical phenomenon, recent depth-electrode recordings suggest that intrinsic subcortical epileptogenesis may arise within the hamartoma, and then spread to the surrounding cortex, including the frontal and temporal lobes, giving rise to secondary epileptogenic foci (13).

Previously, craniotomy was the first choice for HH treatment (14), but this was only associated with a seizure-free rate of 15–54%. Surgery is rarely successful unless the entire lesion is completely removed, and severe perioperative complications can occur, including infarction, diabetes insipidus (DI), electrolyte disorder, appetite stimulation, and memory deficit (4, 15). These complications are more common and severe in giant HHs. In recent years, laser interstitial thermal therapy (LITT) (16, 17), MRI-guided stereotactic radiofrequency thermocoagulation (SRT) (18) and stereo electroencephalography (SEEG)-guided radiofrequency thermocoagulation (RF-TC) (19) have been introduced for HH ablation. While these minimally invasive treatments have improved seizure outcomes, the ablation volume is limited, with these techniques, especially for RF-TC; typically, the RF-TC ablation diameter is ~3–6 mm around the electrode. LITT can achieve lager ablation volumes (10–20 mm diameter). Although the efficacy of HH treatment has been greatly improved due to rapid advancements in surgical technologies, treating giant HHs remains difficult, and many patients require multiple operations (20). In light of these challenges, we designed the high-density focal stereo-array technique to expand the range of thermal coagulation and improve clinical outcomes. We have utilized SEEG-guided RF-TC as our primary procedure to treat giant HH and achieved good clinical outcomes. In this study, we present our preliminary experiences with SEEG-guided RF-TC to treat pediatric patients with giant HH. To date, this report represents the first and largest case series of pediatric giant HH cases treated with SEEG-guided RF-TC.

## Materials and Methods

### Patients

Six consecutive patients (three males and three females; average age: 5.08 ± 4.73 years) with a confirmed HH diagnosis who underwent SEEG-guided RF-TC at Children's Hospital of Fudan University (Shanghai, China) between November 2017 and April 2019 were enrolled in this retrospective study. All patients had HH manifesting as seizures with or without precocious puberty. No cases of HH-associated epilepsy without gelastic seizures enrolled in our case series. Giant HH was defined as masses with a maximum diameter >30 mm (8, 21, 22). This study was approved by the Medical Ethics Committee of Children's Hospital of Fudan University. Written informed consent to participate in this study was obtained from all legal guardians.

### Presurgical Examination and Electrode Implantation

**Figure 1:**
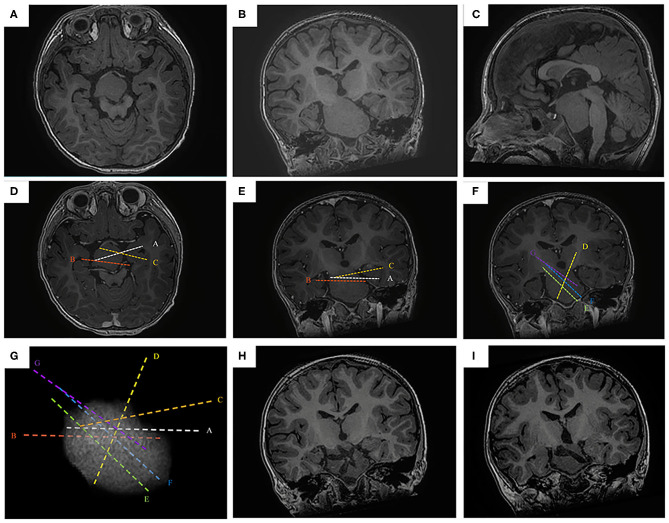
The SEEG-guided RF-TC in a giant HH with wide pedicel attached to hypothalamus. The preoperative MR images (**A**, axial image; **B**, coronal; **C**, sagittal) show a giant, wide pedical HH with left-sided dominant attachment to the hypothalamus (case No. 1). The electrodes are designed to cover the interface in the pedicel (**D**, axial image; **E**, coronal; **F**, sagittal). **(G)** the 3D demonstration of the electrode trajectory, the highest electrode density is at the base of HH. The postoperative MR images obtained 1 year after RF-TC (**H,I**, coronal image) shows the coagulated lesion at the base of the hamartoma. Note that the hamartoma was not coagulated totally, but focuses on the junction between the hamartoma and the hypothalamus.

**Figure 2:**
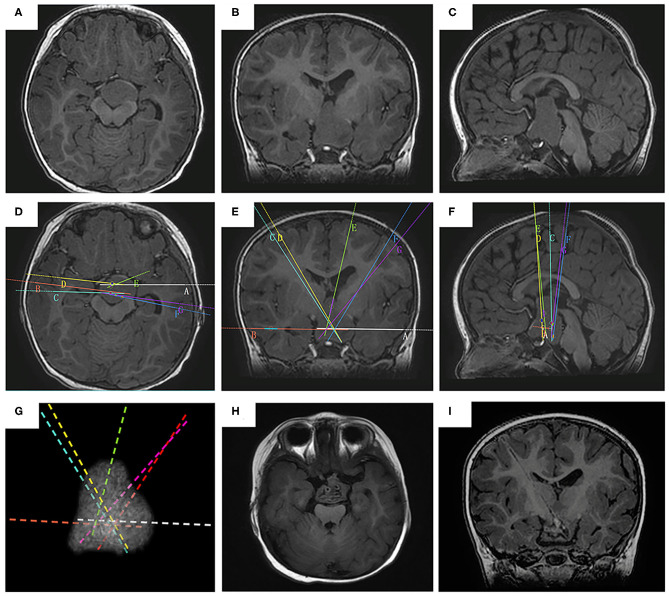
The SEEG-guided RF-TC in a giant type IV HH The preoperative MR images (**A**, axial image; **B**, coronal; **C**, sagittal) show a giant, bilaterally attached HH seat intra- and extra-third ventricle (case No. 2). The electrodes are designed to cover the whole interface (**D**, axial image; **E**, coronal; **F**, sagittal). **(G)** the 3D demonstration of the electrode trajectory. The postoperative MR images obtained 2 months after RF-TC (**H**, axial image; **I**, coronal) shows the coagulated lesion at the base of the hamartoma.

**Figure 3:**
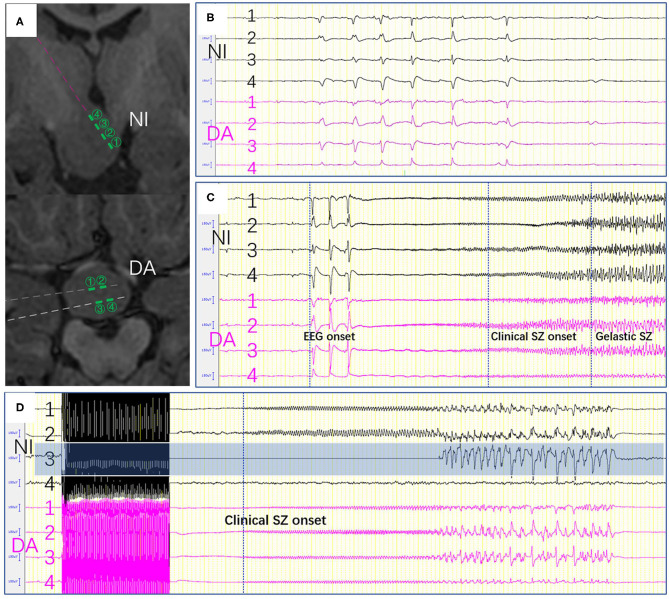
**(A)** The contacts near the interface and in deeper area were shown. **(B)** Epileptic activity between the electrodes near the interface and those in deeper area. **(C)** Seizure onset was recorded in both near the interface and those in deeper area. **(D)** Stimulation-induced seizures were only elicited on one channel (NI3) which located in the interface of HH. SZ, seizure, (NI, near the interface; DA, deeper area).

All patients underwent a comprehensive evaluation that included neuroimaging, in which a 3.0 T MRI with a three-dimensional (3D) T1-weighted sequence (1 mm, including no-contrast and double-contrast sequences) and a 3D coronal FLAIR sequence (1 mm) with fat suppression were obtained as well as long-term video scalp electroencephalography (EEG). Based on the information obtained, strategies for electrode implantation were designed after multidisciplinary discussions. All HH patients with seizures underwent SEEG implantation following a previously described protocol with modifications (23). We used double contrast MRI to display blood vessels instead of the invasive O-arm angiography. Imaging data sets are processed with MRIcron (www.mccauslandcenter.sc.edu/mricro/mricron), Brainsuit (www.brainsuite.org) and Amira (Visual Image, Australia). The key point of the RF-TC strategy is to achieve sufficient coagulation of the interface of the hamartoma with the hypothalamus. The trajectory of the coagulation electrodes was set to pass through the interface using 3D image planning software (Sino Plan, Beijing, China). According to the shape and location of the hamartoma, we designed the electrode implantation scheme as: (1) for giant HHs with broad interfaces attached to the floor of the third ventricle, the electrodes were designed to disconnect the pedicel in a stereo-manner (Figure 1); (2) for giant HHs which extend into the third ventricle and interpeduncular cistern, the high-density stereo-array electrodes were designed to coagulate the interfaces between the lesion and hypothalamus (Figure 2); (3) the trajectories were planned both obliquely and orthogonally: the oblique trajectories were bilaterally designed from the frontal lobe to the lesion; and the orthogonal trajectories were designed from temporal lobe to the lesion; the oblique and orthogonal electrodes formed a three-dimensional array. RF-TC was performed between two adjacent contacts of the same electrode, and also between adjacent contacts of different electrodes to maximally disconnect attachment of the hamartoma. The depth electrodes (Alcis, Besancon, France) were implanted into the HH with the patient under general anesthesia. The complete procedure was performed under the guidance of a frameless robot (Sino-precision, Beijing, China). The robot system is registered using the fiducials, and an error measurement <0.5 mm is acceptable. After the robotic arm automatically reaches the designated position, a drill is used to enter the skull and a coagulation electrode is inserted to open the dura through the robotic guidance instrument. A guidance screw is fixed into the skull. A stylet measured to the depth of the target is then inserted to initiate the electrode trajectory. Then the suitable electrode is implanted through the guidance screw and is secured to the screw at its final position.

### Post-implantation Assessment

Before thermal coagulation, post-implantation assessments including: (1) the exact contact location observed using postoperative 3D computed tomography scan, which was co-registered with the preoperative 3D T1-weighted MRI; (2) intracranial video EEG was performed to monitor both ictal and interictal activity; (3) seizure foci mapping during intracranial video EEG recording.

### RF-TC

The electrode contacts within the HH were selected for RF-TC. This process involved gradually increasing the coagulation power from 0 to 3.5 W over 15 s, holding the coagulation power at 3.5 W for 45 s, which was performed under general anesthesia. A contiguous bipolar RF-TC using one electrode was considered to be one-dimensional, while RF-TC using coupling contacts from two electrodes in one plane was two-dimensional. Three-dimensional cross-bonding of various electrode contacts provided a novel method to optimize SEEG-guided RF-TC (24). Through this process, the scope of thermal coagulation was expanded. According to the intracranial EEG data, the active epileptic discharge sites were coagulated twice with emphasis. Observations were made for at least 24 h after the coagulation process to ensure the absence of residual pathological activity within the area covered by the SEEG.

### Follow-Up

Follow-up included recording seizure outcomes according to Engel's classification (25), as well as EEG and MRI through telephone calls and outpatient visits.

## Results

### Demographics

A summary of the characteristics and results of the children who underwent SEEG-guided RF-TC is presented in Table 1. One patient had previously undergone open surgery. Five patients had precocious puberty, four had gelastic sezures, one had gelastic and tonic seizures, one had gelastic and generalized tonic-clonic seizures. The number of implanted electrodes ranged from 3 to 7, with an average of 5.33. There were no cases of non-epileptic HH in this study.

**Table 1:** Clinical characteristics, SEEG guided RF-TC related events and outcomes of six patients.

**Case No**.	**Sex**	**Age at RF-TC (years)**	**Previous treatment**	**Hamartoma size (mm)**	**Clinical manifestation**	**Seizure frequency**	**Number of electrodes**	**Engel classification**	**Follow-up time (months)**
1	M	1.6	No	43 × 38 × 31	GS and PP	10–20/d	7	I	21
2	F	1.5	No	32 × 29 × 23	GS and TS and PP	5–10/d	7	II	11
3	F	4	No	35 × 22 × 20	GS and PP	30–50/d	5	I	20
4	F	10	Open surgery	32 × 17 × 15	GS and GTCS	10–20/d	3	II	27
5	M	12	No	32 × 22 × 15	GS and PP	3–5/d	5	I	24
6	M	1.4	No	31 × 18 × 16	GS and PP	2–5/d	5	I	18

*GS, gelastic seizures; PP, precocious puberty; TS, tonic seizures; GTCS, generalized tonic-clonic seizure*.

### SEEG Findings

Interictal spikes and spontaneous seizures were recorded from the hamartoma in all the six patients. Most of the time, tonic seizures or generalized tonic-clonic were observed during clusters following gelastic seizures. All of these seizures were the habitual seizures for the individuals. Acute electrical stimulation of the hamartoma could reproduce gelastic seizures in all six patients, when using high frequency parameters (50 Hz), whereas other seizure types were not elicited (Table 2). All patients can record interictal discharges near the interface and in deeper area of the hamartoma. One patient had seizure onset in deeper area, but stimulation-induced seizure was elicited near the interface. One patient had seizure onset near the interface, but stimulation-induced seizure was elicited in deeper area. Four patients had seizure onset recorded both near the interface and in deeper area, among whom including stimulation-induced seizures elicited both near the interface and in deeper area (*n* = 1), only in deeper area (*n* = 1), only near the interface (*n* = 2) (Figure 3 and Table 3). The contacts where the spontaneous seizure detected and stimulation-induced seizures observed are concordant in 4 patients, whereas disconcordant in 2 patients. Moreover, the number of contacts that can induce a seizure is less than the number of contacts that can detect the onset of the seizure.

**Table 2:** The SEEG findings in the six patients.

**Case No**.	**Interictal spike**	**Habitual seizures**	**Spontaneous seizures monitored**		**Electrically induced seizures**	
			**GS**	**Other types**	**GS**	**Other types**
1	_+_	GS	+	–	+	–
2	+	GS and TS	+	+ (TS)	+	–
3	+	GS	+	–	+	–
4	+	GS and GTCS	+	+ (GTCS)	+	–
5	+	GS	+	–	+	–
6	+	GS	+	–	+	–

*GS, gelastic seizures; TS, tonic seizures; GTCS, generalized tonic-clonic seizure*.

*+, present; –, absent*.

**Table 3:** The SEEG finding in the different parts of the HHs.

	**Case 1**	**Case 2**	**Case 3**	**Case 4**	**Case 5**	**Case 6**
**Interictal spike**						
DA	+	+	+	+	+	+
NI	+	+	+	+	+	+
**Ictal onset**						
DA	+	+	–	+	+	+
NI	+	+	+	+	–	+
**Stimulation-induced seizures**						
DA	+	–	+	+	–	–
NI	+	+	–	–	+	+

*DA, deeper area; NI, near the interface; +, present; –, absent*.

### Seizure Outcomes

Seizure outcomes were analyzed in all children for at least 11 months of clinical follow-up. The average duration of follow-up was 20.17 ± 5.49 months. All patients achieved an obvious decrease in seizure frequency. Four patients had Engel I classification outcomes (free from disabling seizures) and two had Engel II classification outcomes. Tonic seizures disappeared but gelastic seizures continued in patient No. 2, while gelastic seizures disappeared but generalized tonic-clonic seizures continued in patient No. 4. However, all residual seizures showed significantly decreased frequency in both patients.

### Complications

We analyzed the records for evidence of any SEEG or RF-TC-related complications, including infection, hemorrhage, medical complications, and hardware-related complications (8). Based on postoperative computed tomography imaging, no patients demonstrated obvious hemorrhages. No hyperthermia, memory impairment and weight gain were observed in our case series. One patient with Delalande type IV (No. 2) who received RF-TC, suffered from syndrome of inappropriate antidiuretic hormone of triphasic episode. The patient had polyuria (6 mL/kg/h), and plasma sodium was 163 mmol/L (reference range: 135–145). Desmopressin acetate (0.1 μg/kg/day, melt form) treatment was started for DI. The patient developed hyponatremia starting on postoperative day four, which gradually worsened. The plasma sodium was decreased to 143 mmol/L, and the initial management included fluid restriction and cessation of desmopressin treatment. Despite fluid restriction for 4 days, the patient's blood sodium levels continued to decrease to 118 mmol/L. Hypertonic saline therapy (3% saline to raise serum sodium by 10 mEq/L) was also added due to the persistence of hyponatremia. One week later, resistant hyponatremia resolved by fluid restriction and hypertonic saline support. Moreover, desmopressin treatment was restarted because of the development of transient DI after RF-TC. The patient recovered well, and no desmopressin therapy was required during the last follow up.

## Discussion

HH is a rare congenital disorder characterized by precocious puberty, seizures, and mental retardation (26). Traditionally, craniotomy has been the first choice for managing HH (14, 27). However, with a surgical approach, it is difficult to achieve sufficient treatment of deep-seated HHs that are surrounded by critical structures. Additionally, craniotomy was typically associated with incomplete resection (28) and multiple complications, including DI, appetite stimulation, and severe surgery-induced injuries (14). Gamma knife surgery (GKS) is often used as well (29, 30). The disadvantages of radiosurgery are its delayed action (31); moreover, large or giant HHs are not appropriate for GKS (30, 32). Several other studies have reported the use of vagus nerve stimulation in HH patients; however, none of these patients achieved seizure-free status (33, 34). Recently, LITT (35), MRI-guided SRT (8, 18), and SEEG-guided RF-TC (19) have been introduced for HH ablation.

Although laser ablation is a safe and effective alternative treatment for gelastic seizures related to HH, the treatment for giant HH is still challenging. A recent study reported that two Delalande type IV hamartoma patients failed to achieve seizure freedom after laser ablation (16). MRI-guided SRT is another option for treating HH. Kameyama et al. reported freedom from GS was achieved in 86.0%, freedom from other types of seizures in 78.9%, and freedom from all seizures in 71.0% in 100 cases (18). Shirozu et al. reported 81.3% of patients were free from GS and 58.3% of them were free from non-GS in 16 giant HH (8). However, repeated surgeries are needed in more than 30% patients with 2–4 repeated SRTs. In rare cases, seizures can occur in patients with non-epileptic HH (36), in which LITT or MRI-guided SRT could result in mistreatment. In the cases of HH-associated epilepsy without gelastic seizures, caution must be taken when considering direct hamartoma surgery. Besides, the high medical cost of LITT hinder its application, it is mainly used in the United States, whereas availability outside the United States remains limited. SEEG-guided RF-TC is another promising method ensuring minimal invasion compared with traditional treatments and could avoid false ablation of a non-epileptic HH (19). In our study, interictal spikes and habitual seizures were recorded from the hamartoma in all patients, which suggests the intrinsic epileptogenicity of the HHs. Electrical stimulation of the hamartoma could reproduce gelastic seizures, but not other seizure types. It is well-known that disconnection at the area close to the interface is critical for better seizure outcomes. Most of the electrodes designed for disconnecting the attachment of the HHs are located near the interface. Due to the small sample size, we have not been able to determine the subcentimeter targets for ablation like that in fMRI (37). There was no difference of epileptic activity between the electrodes near the interface and those in deeper area. Delalande and Fohlen proposed the important concept of disconnection between the lesion and the hypothalamus for preventing the direct propagation of GS. Therefore, the contacts near the interface between the lesion and hypothalamus may be more critical than that in other parts of HHs. However, we could not prove this concept due to the limitation of the number of electrodes as well as small case numbers. We observed that tonic seizures disappeared in patient No. 2, while generalized tonic-clonic seizures did not disappear in a 10 years-old patient (No. 4). These findings suggest the existence of a dynamic ictal network organization, with the possibility of “kindling-like” relationships between the HH and the cortex. In some older patients, independent stage epileptiform activity in the secondary epileptogenic area persisted after removal of the primary focus without running down. Some researchers have advocated that a short time window (<10 years) from epilepsy onset to surgical treatment of HH seems to be crucial to prevent the independent stage and cure epilepsy (38).

The main weakness of SEEG-guided RF-TC is the limited range of thermal coagulation. To solve this problem, we used dense stereotactic electrode array implantation to cover the lesions, especially sites where the hamartoma base was connected with normal structures. On the basis of the single electrode thermocoagulation, 3D cross-bonding of contacts between electrodes was further used to maximally destroy the hamartoma, especially the pedicles of hamartoma attachment, which had good effects. Using this method, we were able to fully record the discharge in the hamartoma and destroy it fully in one stage, with good efficacy. If the patients have residual seizures, additional surgery could be applied (20). As shown in Figure 2, this patient had residual gelastic seizure and incomplete disconnection may be the cause of residual epilepsy. The exploration of SEEG and the extent of ablation were restricted by the number of electrodes. Even though high-density electrodes were used, the electrodes could not cover the entire interface. Hence, this procedure may result in incomplete disconnection, then repeated surgery is indicated. That might be one of the limitations of the procedure using SEEG. In some cases, trajectories were designed to transversely disconnect the HH attachment from the third ventricle. These trajectories are risky for passing through the basal cistern from the temporal lobe to HH. Special attention should be paid to this point. It needed to be made adjustments according to the vascular anatomy.

Because giant HHs are often so large and closely connected to important structures of the hypothalamus, there is still a chance for complications, including DI and hyponatremia, but these complications were transient and did not result in long-term impairment of the patients' quality of life. As most of our patients were younger, recovery may be better.

## Conclusion

SEEG-guided RF-TC is a unique and promising technique that can be used to directly manipulate HH based on both SEEG and MRI signals. SEEG could show ictal activity of gelastic seizures in HH. Although the exploration of SEEG and the extent of ablation were restricted by the number of SEEG electrodes, ablation using multiple SEEG electrodes enabled maximum ablation. One-stage high-density focal stereo-array SEEG-guided radiofrequency was safe and effective for treating pediatric giant HH patients. It can be an alternative method to treat giant HHs where LITT is unavailable.

## Data Availability Statement

All datasets generated for this study are included in the article/supplementary material.

## Ethics Statement

The studies involving human participants were reviewed and approved by the Medical Ethics Committee of Children's Hospital of Fudan University. Written informed consent to participate in this study was provided by the participants' legal guardian/next of kin. Written informed consent was obtained from the individual(s), and minor(s)' legal guardian/next of kin, for the publication of any potentially identifiable images or data included in this article.

## Author Contributions

MW and YZho: study concept and design, data collection and analysis, drafting and revision of manuscript, and full responsibility of data. YZha and WS: data collection, analysis, and manuscript revision. SZ and YW: data collection and statistical analysis. HL and RZ: study concept and design, critical revision, and review of final manuscript. All authors contributed to the article and approved the submitted version.

## Conflict of Interest

The authors declare that the research was conducted in the absence of any commercial or financial relationships that could be construed as a potential conflict of interest.
